# The Role of Surgery in Lung Cancer Treatment

**DOI:** 10.3390/cancers12102777

**Published:** 2020-09-28

**Authors:** Francesco Petrella

**Affiliations:** 1 Department of Thoracic Surgery, IRCCS European Institute of Oncology, 20141 Milan, Italy; francesco.petrella@unimi.it; 2 Department of Oncology and Hemato-Oncology, University of Milan, 435–20,141 Milan, Italy

Lung cancer is the leading cause of cancer-related death accounting for 1.6 million cancer-related deaths worldwide every year [1]. It is a heterogeneous disease and, for this reason, therapies need to be personalized according to the performance status of the patient, extent of disease, histology and biology of the tumour. Primary pulmonary tumours are usually divided into three categories, according to the best treatment available: early stage or resectable lung cancer, locally advanced and metastatic disease [1].

Patients suffering from resectable lung cancers are standardly treated by lobectomy and resection of mediastinal lymph-nodes; this procedure can be performed by thoracotomy or by a video-assisted or robotic-assisted approach in order to minimize chest-wall incisions and reduce the postoperative length of stay [2]; anyway, the cornerstone of surgical treatment of NSCLC remains radical resection of the tumour and adequate lymph-node dissection; eligibility for a minimally invasive approach depends on several factors like the tumor’s volume and location, clinical history of the patient and the expertise of the center with the selected approach [3,4].

Locally advanced lung cancer belongs to the stage III and multimodal approach (chemotherapy, surgery, radiotherapy and—more recently—immunotherapy and biologic therapy) as the gold-standard treatments.

Although many centers do not consider surgical resection an option for stage III patients, selected IIIA patients can be eligible for surgical resection as an additional tool to other treatment options; anyway it should be clearly taken into consideration that there are higher intra- and postoperative risks, thus suggesting this option only in very experienced surgical centers [5,6,7].

Advanced stage or metastatic lung cancer patients (stage IV) can be treated—according to their mutational status and tumour burden—by tyrosine kinase inhibitors (TKIs), immunotherapy or by standard first-line treatment with platinum-based doublet chemotherapy in the case of unknown mutational status or without known driver mutation [8,9,10]. This group of patients has maximally benefitted from the recent significant improvements in new drugs developments and apparently there is no curative role for surgery in this subset of patients. Anyway, it should be clearly taken into consideration the contribution that surgery may offer at this stage to control symptoms, improve quality of life and prevent life-threatening events. There are, in fact, several scenarios where palliative surgical procedures can be useful to overcome dangerous complications that are more frequently observed because of the increment of the overall survival in metastatic lung cancer patients. Pleuro-peritoneal window or pleuro-pericardial window—for instance—can effectively treat pericardial effusions thus preventing cardiac tamponade; similarly talc poudrage by a video-assisted thoracic approach can treat recurrent and symptomatic pleural effusions; operative rigid bronchoscopy can be used to treat bleeding airway obstruction by laser-assisted mechanical resection or by airway stents.

In conclusion although the ideal setting for surgical treatment of lung cancer is the early-stage resectable disease, a significant role may be played in locally advanced disease within a multimodal approach in carefully selected patients and in stage IV patients to treat or prevent severe and life-threatening conditions.
